# Clinical management of borderline tumours of the ovary: results of a multicentre survey of 323 clinics in Germany

**DOI:** 10.1038/sj.bjc.6605065

**Published:** 2009-05-12

**Authors:** A Coumbos, J Sehouli, R Chekerov, D Schaedel, G Oskay-Oezcelik, W Lichtenegger, W Kuehn

**Affiliations:** 1 Outpatient Practice of Gynecological Oncology, Albrechtstr. 48, 12167 Berlin, Germany; 2 Department of Gynecology, European Competence Center for Ovarian Cancer, Campus Virchow, Charité University Hospital, Augustenburger Platz 1, 13353 Berlin, Germany; 3 Laser- und Medizin-Technologie GmbH, Fabeckstr. 60-62, 14195 Berlin, Germany; 4 Department of Gynecology and Gynecologic Oncology, Campus Benjamin Franklin, Charité University Hospital, Hindenburgdamm 30, 12203 Berlin, Germany

**Keywords:** borderline ovarian tumour, clinical management, diagnosis, therapy, multicentre survey

## Abstract

The aim of this survey was to analyse the standard of care in diagnostic, surgery, chemotherapy and aftercare management for patients with borderline tumours of the ovary (BOTs) in Germany. A structured questionnaire comprising different dimensions was sent to all 1114 gynaecological departments. The questionnaire could be returned anonymously. The overall response rate was 29.0% (323 departments). Most departments were on secondary care (71.8%), tertiary care (23.2%) or university hospital (5.0%) level. Most clinicians performed not more than five BOT operations (89.2%) per year. Most departments (93.2%) used in addition to classical bimanual examination and vaginal ultrasound, tumour marker CA-125 detection, CT scan, MRI or PET-CT techniques. Departments in university and tertiary care hospitals performed more often a fresh frozen section (87 *vs* 64%). In young women, clinicians performed much seldom unilateral salpingo-oophorectomy (92%) and only in 53% biopsies of the contralateral ovary. Generally, biopsies of the contralateral ovary were performed in 4–53% of the patients. Chemotherapy was mostly favoured in ‘high-risk’ patients with tumour residual, microinvasion or invasive implants. Thus, a high grade of insecurity in diagnostic and therapy of BOT exists in some gynaecological departments and underlines the need for more educational and study activities.

Borderline tumours of the ovary (BOTs) are a specific tumour entity that represents some characteristics of malignant ovarian tumours, but which does not show any destructive stromal invasion. Thus, the borderline tumour – in contrast to that of an invasive ovarian carcinoma – has generally had an excellent prognosis (Sehouli *et al*, 2005a, 2005b; Tinelli *et al*, 2006; Skirnisdottir *et al*, 2008). Borderline tumours of the ovary constitute 8–10% of all ovarian tumours. Having an incidence of 1.8–4.8 out of 100 000 women per year, they belong to the seldom ‘non-benign’ epithelial ovarian tumours (Hart, 2005; Tinelli *et al*, 2006; Wong *et al*, 2007). In contrast to the case of ovarian carcinomas, most of the patients are diagnosed at an early stage I (Gotlieb *et al*, 2005; Yokoyama *et al*, 2006; Cusido *et al*, 2007). Today no specific diagnostic method is able to discriminate reliably between an early carcinoma of the ovary and a borderline tumour (Seidman *et al*, 2004; Hart, 2005; Sehouli *et al*, 2005a, 2005b; Kurman *et al*, 2008). For many years, BOT of the ovary was considered to be a pre-malignant disease. On the basis of clinical, pathological and molecular genetic studies, different new models of ovarian carcinogenesis are being established (Sherman *et al*, 2004; Denkert and Dietel, 2005; Meinhold-Heerlein *et al*, 2005; Kurman *et al*, 2008). According to Denkert and Dietel (2005), G1 ovarian carcinomas originate step by step from a cystadenoma or a borderline tumour. In contrast to this, moderate (G2) and poorly differentiated (G3) ovarian carcinomas seem to result from a ‘*de novo* synthesis’ of a highly malignant tumour, which skips these ‘developmental steps’. Kurman *et al* (2008) differ also between type I tumours presenting slow growing, confined to the ovary and most originating from borderline tumours. Type II tumours are rapidly growing, highly aggressive neoplasms characterised by TP53 mutations and a high level of genetic instability.

The detection of extraovarian invasive implants determines to a great extent the prognosis of borderline ovarian tumours (Denkert and Dietel, 2005; Sehouli *et al*, 2005a, 2005b). Usually, in most patients with BOT, non-invasive implants are common, whereas 6% of the women present invasive implants, which are strongly associated with a poorer prognosis (Longacre *et al*, 2005). In the clinical management, the observation of non-invasive implants appears to have no influence on survival or on any clinical consequence (Jones, 2006; Rollins *et al*, 2006; Silva *et al*, 2006; Chang *et al*, 2008).

Although most part of BOT therapy was strongly oriented towards the ovarian cancer treatment (Suh-Burgmann, 2006; Tinelli *et al*, 2006; Cadron *et al*, 2007), adjuvant systemic chemotherapy is nowadays not generally indicated (Boran *et al*, 2005; Sehouli *et al*, 2005a, 2005b; Romagnolo *et al*, 2006; Wong *et al*, 2007; Yinon *et al*, 2007).

In contrast to ovarian cancer (du Bois *et al*, 2001, 2005), no surveys have yet been carried out in regard to the clinical management of BOT in Germany.

A structured survey about the current clinical management of BOTs is deemed absolutely necessary to gain new approaches in the conception of prospective trials and to identify the demand for further education and research activities concerning the topic of BOT.

## Materials and methods

Over a period of 12 months, a questionnaire concerning the therapy management of BOT was sent by conventional post to a total of 1135 clinics in Germany. These included all university hospitals, hospitals of tertiary or secondary medical care and public community general practitioners' clinics (a special subtype of secondary care institutions in Germany entailing considerable involvement with the general practitioners in the inpatient management). The mail questionnaires were sent directly to the heads of the department of obstetrics and gynaecology of each institution.

The questionnaire was developed in a multistep process according to similar surveys (Sehouli *et al*, 2005a, 2005b). First, relevant topics and a list of questions were formulated by an expert group including gynaecological oncologists, pathologists and statisticians (AC, JS, DS and WK) on the basis of a workshop and according to the national and international guidelines (German Society of Obstetrics and Gynecology and the National Cancer Institute). The conceived catalogue of questions was finally converted into a structured questionnaire. This focused on statistical data, such as clinical structure and clinical size of the institutions, number of clinics that were performing surgical and adjuvant treatment for ovarian and borderline tumours, with the special focus on diagnosis, therapy, prognosis and follow-up aspects of BOT. A special consideration was the management of patients who had desired to preserve fertility. All possible answers and combinations were provided in a multiple-choice manner.

Before beginning the multicentre survey, the questionnaire, which included 25 questions, was checked by 20 volunteers, all medical personnel, for comprehensibility and reproducibility in a pilot study. Here, the average time needed to fill it out was 11 min (range 6–15 min).

A written consent was obtained by the ethics committee of the Charité – University Hospital.

For statistical evaluation, we used SPSS for Windows (version 14.0 SPSS, Chicago, IL, USA). The present investigation was carried out for the generation of hypothesis. For this, all analyses were worked out in a primarily descriptive manner. The *U*-test of Mann and Whitney, and alternatively the *χ*^2^-test, was used for the control of significances of unbound random samples. For a level of significance, the limit was set to 5% (*P*<0.05).

## Results

Of the 1135 clinics that were addressed, 21 had no special department of gynaecology and were therefore excluded from the evaluation. Of the remaining 1114 clinics, 328 answered the questionnaires. This corresponds to a response rate of 29.4%. Only 5 (0.4%) of the questionnaires were not eligible for evaluation as their answers were either incomplete or unreadable. Out of the 30 university hospitals in Germany contacted, 16 (53%) took part in the survey.

### Clinic structure and clinic size (number of gynaecological beds)

From all of the questionnaires returned, 5% came from university clinics (*n*=16), 23% from tertiary care hospitals (*n*=75%), 65% from secondary care hospitals (*n*=209) and 7% from general practitioners' clinics (*n*=23).

With respect to the size – that is the number of beds in the gynaecological departments – the following picture was observed (one clinic revealed no data): 78 clinics (24%) had a maximum of 30 beds, 155 clinics (48%) had 31–50 beds, 81 hospitals (25%) had 51–100 and only 8 institutes (3%) had more than 100 gynaecology beds.

### Number of ovarian cancer operations

Responding to the question ‘How many patients with ovarian cancer have you treated in the past 12 months by surgery?’, 5% answered that they had not undertaken any such surgical interventions. The majority of the departments (*n*=270, 80%) reported no more than 20 ovarian carcinoma operations per year. Within 1 year, almost half of the institutions (49%) had done 1–10 and one-third (35%) 11–20 ovarian carcinoma operations, whereas 17 clinics (5%) treated 21–30 ovarian carcinomas, and only 20 hospitals (6%) had undertaken more than 30 interventions per year.

According to the size of the academic departments of gynaecology and obstetrics, 69% (11 out of 16) treated more than 30 patients with ovarian cancer by surgery and 25% (4 out of 16) not less than 11 patients per year. The majority of the tertiary care hospitals (65%) treated 11–30 ovarian carcinomas each year. Only 11% (8 out of 75) of these clinics treated more than 30 women with ovarian cancer and 24% (18 out of 75) up to 10 women by surgery in 1 year. The majority of the secondary care hospitals (59%) treated no more than 10 ovarian carcinomas per year. In 33% (69 out of 209) of these clinics, 11–20 carcinomas were treated surgically, in 4% (8 out of 209) 21 or more, and in only 3% (7 out of 29) no ovarian carcinomas were treated operatively at all. Public general practitioners' clinics treated ovarian carcinomas more seldom than did all other institutions. In 15 of the 23 clinics of this type (65%), no more than 10 ovarian carcinomas were operated on, whereby 8 of the hospitals (35%) did not perform any ovarian cancer surgery at all.

**Figure 1:**
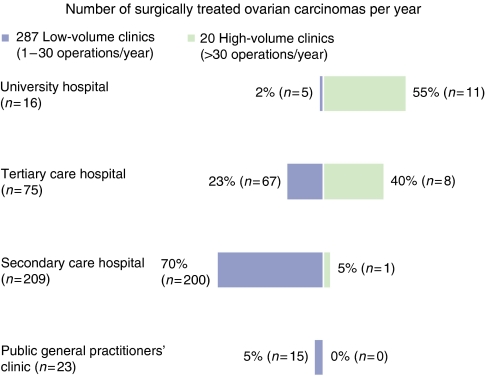
Hospital category and number of surgically treated ovarian carcinomas per year (*n*=323, 1 clinic not specified, 15 clinics did not perform any operations within the time of evaluation, *P*=0.0001).

Thus, 287 of the 323 hospitals participating in the survey were ranked as ‘low-volume clinics’, with 1 to maximal 30 operations per year, and only 20 institutions as ‘high-volume clinics’, with more than 30 operations per year (Figure 1). Within the ‘high-volume clinics’, 95% were university and tertiary care hospitals. We here detected significantly more surgical interventions in women with ovarian carcinomas (*P*<0.05).

### The number of surgically treated BOTs

As to the question ‘How many BOTs have you operated on in the last 12 months?’ 157 of the replies (48.6%) reported a maximum of 2 and 131 (40.6%) reported 3–5 borderline tumour operations. From the 323 participants, 30 (9%) treated 6–10 BOTs by surgery per year and only 3 departments (0.9%) more than 10. Two clinics (0.6%) submitted no statements.

Analysing the number of surgical interventions per year with respect to the clinic type, we observed that 31% (5 out of 16) of the university hospitals performed 3–5 BOT operations, 56% (9 out of 16) 6–10 and 6% (1 out of 16) more than 10 BOT operations per year. One department reported a maximum of two operations of borderline tumours each year.

Among the tertiary care hospitals, we encountered the following situation: 56% (42 out of 75) of the clinics operated within 1 year 3–5 BOTs, 16% (12 out of 75) 6–10 and only one clinic (1.3%) more than 10 borderline tumours. The majority of the secondary care hospitals (57%) treated less than 2 BOTs a year, whereas 80 departments (38%) performed 3–5 and 10 (5%) more than 6 BOT operations per year. Of the 23 public general practitioners' clinics, 78% (18 out of 23) undertook an operative treatment of maximal 2 borderline tumours and 17% (4 out of 23) 3–5 operations per year.

**Figure 2:**
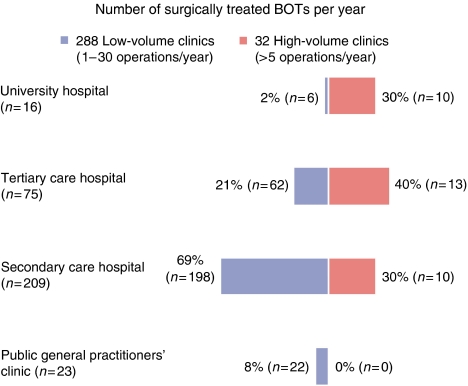
Hospital category and number of surgically treated BOTs per year (*n*=323, 2 clinics not specified, *P*=0.0001).

The analysis of the operation volume – ‘high-volume clinics’ (>5 BOT operations per year) compared with ‘low-volume clinics’ (1–5 BOT operations per year) with respect to the clinic size – showed that 30% of the BOT operations were performed at university institutions, 40% at tertiary care hospitals and 30% at secondary care hospitals (Figure 2). Thus, statistically seen, most patients operated on BOT were treated in university and tertiary care clinics than in all of the smaller clinics (*P*<0.05).

### Pre-operative diagnostics

When asked the question ‘Which pre-operative diagnostics were performed at your department in a case of suspicious ovarian tumour?’, 93% (301) of the participants answered that in addition to gynaecological examination and vaginal ultrasound in the case of an unclear ovarian tumour, further diagnostic procedures had to be performed. The most common additional imaging methods used were 76% CT scan, 66% Doppler ultrasound, 36% MRI scan and 1.7% PET-CT scan. One of the most commonly used diagnostic methods (95%) was the detection of tumour markers (CEA und CA-125).

The routine detection of the tumour marker was performed equally often in all participating departments: in 93% of the universities' *vs* 95% of the other clinics (NS). Also, among the use of Doppler sonography, we observed no differences (60 *vs* 67%; NS). In contrast, university clinics performed CT scans much more seldom than did non-university clinics (40 *vs* 78%; *P*=0.003). Also, MRI was applied only in a limited number of cases in university clinics (20 *vs* 37%), although this difference was not significant (NS). The PET-CT scan was generally not applied as an additional diagnostic in the case of supposed ovarian tumours in the universities', but it was in 2% of the non-university clinics (NS).

‘Which was the most common diagnosis suspected in the case of BOT prior to the operation?’

For this question, 54% of the interviewed participants replied that before the operation they diagnosed an ‘unclear ovarian tumour’. For 28% of the interviewers, an ovarian carcinoma, and for 20%, a benign ovarian tumour was suspected. Only in 8.4% of the cases was the pre-operative diagnosis that of a borderline tumour. Some of the clinics (3.4%) did not answer this question. We did not ascertain any statistical differences according to the clinic types observed in the survey.

### Type and radicality of surgical intervention

As to the question ‘Which operation techniques were used for the diagnosis of BOT?’ 48% of the clinicians were found to use conventional laparotomy. Primary endoscopy was the basis for the diagnosis of 15% of the BOTs, whereas 19% used a diagnostic laparoscopy, followed by completion of the operation in a second intervention. A switch from laparoscopy to laparotomy during the primary surgical intervention was performed by 18% of the clinics.

In the university clinics, 53% of the BOT diagnoses occurred after laparotomy and 20% after laparoscopy. In 7%, after diagnostic laparoscopy, the tumour resection was performed by laparotomy in the same session, but 21% preferred a subsequent operative intervention. Tertiary care hospitals (*n*=75) showed a similar surgical management: 53% laparotomy, 12% laparoscopy, 16% diagnostic laparoscopy with a following switch to laparotomy and 18% laparotomy in a second intervention. In secondary care hospitals (*n*=209), 47% of the BOT cases were diagnosed and treated by primary laparotomy, 14% by laparoscopy, 18% by laparoscopic diagnosis and joined switch to laparotomy, and 20% performed laparotomy in a subsequent intervention. Public general practitioners’ clinics (*n*=23) usually preferred the primary laparotomy (40%) followed by primary laparoscopy (26%), laparoscopy with switch to laparotomy (25%) and the uncommon subsequent laparotomy for completion (9%).

In regard to the clinical structure and type here, no significant differences in the surgical procedures were noted (NS).

### Fresh frozen section as an intraoperative diagnostic tool

**Figure 3:**
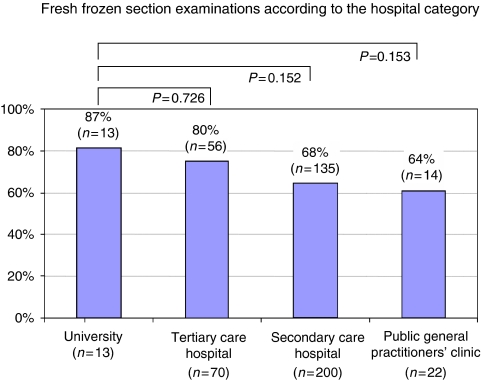
Implementation of the fresh frozen section in the intraoperative diagnosis with respect to the hospital category (*n*=323, 16 clinics not specified).

Regarding the question ‘Did you perform any fresh frozen section intraoperatively to confirm the diagnosis of a BOT?’, 68% of the clinicians confirmed the importance of the intraoperative diagnostic. In 87% of the university clinics, 80% of the tertiary care hospitals, 68% of the secondary care hospitals and 64% of the general practitioners’ clinics, regular fresh frozen examination was the standard for intraoperative diagnosis (Figure 3).

On the basis of the results of the fresh frozen sections, 15% of the clinics performed the complete tumour resection during the same operation. In 56%, this approach led to a subsequent surgical intervention in cases where the primary diagnosis was still not clear. In all, 25% of the clinicians favoured in general a second surgical intervention for completion, whereas 4% did not offer any answer.

### Surgical management in case of BOT

**Figure 4:**
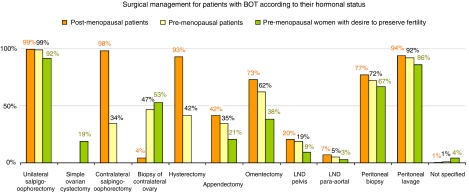
Surgical management for patients with BOT according to their hormonal status (*n*=323, multiple answers are possible).

To evaluate the operative management in case of BOT, we had asked ‘Which procedures would you prefer in the surgical therapy of BOT?’ We here differentiated between three groups of patients: pre-menopausal, post-menopausal and women in the reproductive age, who still desired to preserve fertility (Figure 4).

Concerning the operative procedures taken for pre-menopausal women with a BOT, 99% of all participants performed a unilateral adnexectomy and 34% a bilateral adnectomy. In university clinics, a bilateral adnectomy was performed more often (56%) than in the other clinics: tertiary care hospitals 35%, secondary care hospitals 34% and general clinics 17% (NS). Instead, among practitioners’ non-university clinics, a biopsy from the contralateral ovary was favoured. Hysterectomy was observed in 42% of the clinics and appendectomy in 35%. Omentectomy was performed in 62%, a rate much more strongly preferred at the universities’ clinics (88%, *P*=0.002) than at tertiary care (76%), secondary care (56%) or general practitioners’ hospitals (52%). Peritoneal biopsies were performed in 94% of the university clinics, 83% of the tertiary care hospitals, 70% of the secondary care hospitals and 43% of the general practitioners’ clinics. Samples of peritoneal cytology were collected by 92% of the clinics. A pelvic lymph node dissection followed in 18.9% of the surgical interventions.

For post-menopausal patients, the unilateral adnectomy was performed in 99% of the cases; however, the bilateral adnexectomy was much common here, being used in 98% (*P*<0.05%). Additional hysterectomy (93%) and omentectomy (73%) were also standard procedures. Omentectomy was observed without any exception (100%) in all participating university departments, in 84% of the tertiary care, in 69% of the secondary care hospitals and in 57% of the public general practitioners’ centres (*P*<0.005). Peritoneal cytology followed with a total of 94%, and peritoneal biopsies were collected in 77% of the cases.

For women in the reproductive group with the desire to still have children, the resection of the concerned ovary followed in 92%; even in 19%, a tumour/ovarian cystectomy was performed. The contralateral ovary was usually checked by biopsy by 53% of the participants, but not surgically removed. Much less often were peritoneal biopsy (67%) and peritoneal wash cytology (86%) performed (Figure 4). Omentectomy was performed in 81% of the universities, but only in 57% of the tertiary care units, 31% of the secondary care units and in as few as 17% of the public general practitioners’ centres (*P*<0.05). Peritoneal biopsies were performed in 81 and 80% of the university departments and tertiary care hospitals, respectively, but only in 64% of the secondary care and 39% of the general practitioners’ hospitals.

After the patients have declared the wish to have children, 47% of the clinics recommended a completion of the surgical resection of the involved ovary and/or contralateral adnectomy. Here, no significant differences were found between the various profiles of institutions (NS).

### Risk factors that indicate the necessity of adjuvant treatment of BOT after surgery

For prognostic of the factors that determine a high-risk group of women with BOT who might otherwise benefit from adjuvant treatment, most of the interviewees identified the incomplete tumour resection, the evidence of microinvasive implants and the tumour stage. Seventy-seven percent of the participants stated that the detection of microinvasive implants is associated with higher aggressiveness and a high risk of recurrence, followed by tumour rest (74%) and advanced stage disease FIGO III (52%) or IV (54%).

### Type of adjuvant treatment after primary surgery

Out of all participants, 30% did not recommend an adjuvant treatment after the primary surgery. In most departments (64%), chemotherapy was suggested only in the high-risk situation: tumour residuals, microinvasion with evidence for invasive implants or in mucinous or clear cell histological subtypes. Chemotherapy was generally implemented as an adjuvant treatment by only two clinics (0.6%). Few departments (1.5%) preferred even intraperitoneal (i.p.) chemotherapy, i.p. radionuclide therapy or whole abdominal irradiation as further treatment options. The adjuvant treatment was not preferred in 25% of university clinics (4 out of 16), whereas 12 departments (75%) saw a potential benefit of chemotherapy for high-risk patients. The majority (46) of the 75 tertiary care hospitals (62%) would recommend chemotherapy among risk constellations; the rest of the clinics (33%) would generally not prefer adjuvant treatment for BOT. One tertiary care clinic favoured i.p. chemotherapy as an optimal treatment, whereas only one gynaecological department implemented chemotherapy for all patients with BOT diagnoses. In all, 136 of the 209 secondary care hospitals (65%) considered chemotherapy as indicative for high-risk patients, 59 hospitals (28%) generally recommended no adjuvant indication. One secondary care hospital concluded that chemotherapy was advisable for all patients with BOT after surgery, and three others preferred alternative therapy options: i.p. chemotherapy or whole abdominal irradiation. In 35% of public general practitioners’ hospitals, no adjuvant treatment was recommended, but 48% preferred chemotherapy for high-risk BOTs. Regarding this question there were no significant differences noticed in the treatment management of the institutions participating (NS).

### Type of chemotherapy

As to the question ‘Which type of i.v. chemotherapy would you apply to patients with a BOT?’ 75% said that they favour the conventional treatment with carboplatin and taxanes. Others (6%) would prefer a combination of cisplatin and taxanes. Nineteen percent of the clinicians would treat with carboplatin as a single agent, and 2.5% would use cisplatin as a single agent. In all, 1.5% of the clinicians would search for consultation with another oncological specialist for recommendation.

### Management of patients with BOT recurrence

**Figure 5:**
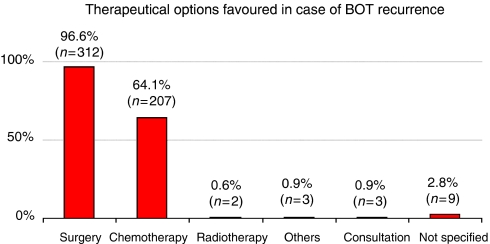
Management of the recurrence of BOT (*n*=323, multiple answers are possible).

To the question ‘Which treatment is indicated for patients with relapsed BOT?’ 97% designated surgical treatment as their first option. Another 64% claimed that chemotherapy was the second best option here. In contrast to this, only 0.6% considered that radiation therapy would be a possible treatment. In all, 0.9% of the interviewees would prefer a consultation with other experts to obtain decision (Figure 5).

In the university as well as non-university clinics, the therapy of choice was the radical tumour resection (100 *vs* 96%). As a second option, participants chose the application of i.v. chemotherapy (50 *vs* 65%); here, no statistically significant differences between university and non-university hospitals were noted (NS).

### Aftercare management

In regard to the inquiry ‘How do you organise the aftercare of patients with BOT?’, most participants specified their recommendation of performing regularly every 3 months a check-up that includes clinical examinations (96%), gynaecological sonography (95%) as well as a tumour marker control (67%). For imaging diagnostic procedures during the aftercare, 28% of the clinicians used a CT scan, 13% an MRI scan and 2% the PET-CT scan. Moreover, 26% of the participants considered the second-look laparoscopy as an adequate method in aftercare management.

**Figure 6:**
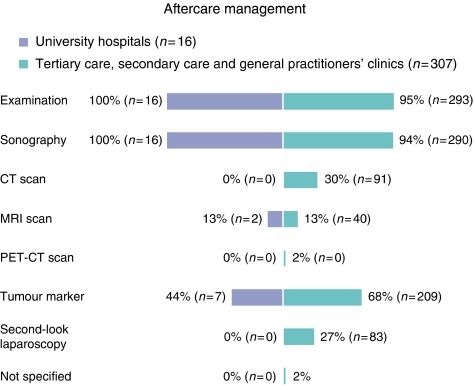
Management of aftercare for patients with BOT, with percentage implementation of diagnostic procedures (*n*=323, multiple answers are possible).

With respect to the management of the aftercare recommendations for women with BOTs, we observed significant differences between university institutions and other clinics (Figure 6) (*P*<0.05). In university clinics, the aftercare includes mostly a gynaecological examination with sonography, and in one section of the departments (44%), the routine tumour marker controls. As opposed to this, all other clinics used CT scan (*P*=0.010), tumour marker control (*P*=0.044) and second-look laparoscopy (*P*=0.016) to a significantly higher degree.

## Discussion

With this survey, we have performed the first representative analysis of actual trends and institutional standards for the clinical management of borderline tumours in Germany.

In the field of pattern of care for women with BOT, only a few multicentre studies have already been published on an international level. Most of these were limited to a monocentric and retrospective analysis of the incidence, treatment procedures and they often present only a small number of patients (Boran *et al*, 2005; Romagnolo *et al*, 2006; Cusido *et al*, 2007; Kumpulainen *et al*, 2007; Skirnisdottir *et al*, 2008).

Within all of the 323 clinical departments analysed, the vast majority treated no more than 30 patients with ovarian carcinoma per year. These data are in agreement with the recently published data of the German Gynecological Oncology Group (AGO) concerning the treatment quality and pattern of care for patients with ovarian cancer (du Bois *et al*, 2005). Most of the clinics reported less than five patients with BOT diagnosis per year. This finding is in line with national and international incidence data, where the ratio of patients with ovarian carcinoma to patients with BOT was between 1 : 10 and 1 : 20 (Romagnolo *et al*, 2006; Skirnisdottir *et al*, 2008).

With 29.0% responses to our survey, we have reached a significant level that allows a representative interpretation of the data pool examined. Also, the data of the German quality assurance survey are based on the data of one-third of the patients treated (du Bois *et al*, 2005). Nevertheless, for any careful interpretation of our results, we should not overlook the facts that there was indeed a limited participation of many hospitals and also the absence of a significant verifiability of all recommendations given to our questions (e.g., absence of control of detailed operation reports, histological diagnosis etc.). Furthermore, we like to underline that we have not monitored the documentation of the investigators.

### Diagnostics

In most cases, ovarian cancer begins asymptomatically, spreading in the peritoneal cavity and will then be diagnosed at an advanced stage during the time of primary diagnosis (FIGO stage III or IV), whereas the BOT is diagnosed mostly in stage I (Gotlieb *et al*, 2005; Sehouli *et al*, 2005a, 2005b; Yokoyama *et al*, 2006). Similar to early carcinomas of the ovary, BOTs are most commonly detected as accidental histopathological findings after primary surgery for benign or suspected ovarian mass (Seidman *et al*, 2004; Jones, 2006; Schem *et al*, 2007; Chang *et al*, 2008). In our study, this applies to 30% of the cases; 28% have their origin in suspected ovarian tumours and 19.5% arise from a primary benign ovarian tumour.

On the basis of the current literature, vaginal ultrasound and possibly additional Doppler ultrasound as well represent the best diagnostic tool for the detection of BOT (Reles *et al*, 1997; Cohen and Fishman, 2002; Togashi, 2003; Schem *et al*, 2007). Results of our survey indicate the need for more adequate staging of borderline tumours in some hospital categories. Especially for our assessment, this involves the often-used CT and MRI examinations. Moreover, the commonly observed tumour marker CA-125 – which is much less enhanced in BOTs than in ovarian cancers – does not suitably improve any positive or negative predictive value of the examination (Hart, 2005; Schmalfeldt and Pfisterer, 2007).

### Surgical therapy

The therapy of BOT leans closely on the clinical management of ovarian carcinoma and is directed at definitive staging, that is complete or maximum tumour debulking, although the treatment of BOTs does show some relevant differences (Cadron *et al*, 2007; Schmalfeldt and Pfisterer, 2007). According to the FIGO classification, the state-of-the-art surgical treatment includes a detailed exploration of the entire abdomen, bilateral salpingo-oophorectomy (BSO), hysterectomy, omentectomy, peritoneal lavage (or ascites sample) and peritoneal biopsies and resection of all suspected lesions (Trope *et al*, 2000; Schmalfeldt and Pfisterer, 2007). For mucinous tumours, an appendectomy should be performed to exclude any ovarian metastasis of possible mucinous tumour of the appendix (Lichtenegger *et al*, 1998; Cadron *et al*, 2007; Schmalfeldt and Pfisterer, 2007).

The accuracy of the frozen section diagnosis of BOT is very limited. In the final histological examination, an estimated 23–27% of all tumours indicated signs of an invasive growth. For mucinous BOTs, this rate seems to be higher than that for serous tumours (Hart, 2005). In our study, 56% of all participants favoured a subsequent interval surgery for those cases where the intraoperative frozen section was not clear. In comparison with other clinics, gynaecological departments on university and tertiary care hospitals performed much more often a fresh frozen section. Specific factors that pre-determine the specific clinical management were not asked for in our survey. Here, infrastructural reasons such as the unavailability of an individual pathological unit or the principal favour of interval surgery in case of BOT may have a substantial impact on the surgeon's decision in the clinical routine.

As there are often young women in the reproductive age diagnosed with a BOT who still desire preservation of fertility, all conservative surgical options must be heeded (Boran *et al*, 2005; Marcickiewicz and Brannstrom, 2006; Yinon *et al*, 2007). Thus, if a BOT has been diagnosed with no invasive implants, after an intensive patient consultation, the following procedure can be chosen: a detailed exploration of the abdomen, a peritoneal lavage, peritoneal biopsies from all regions of the abdomen, a unilateral salpingo-oophorectomy (USO), an omentectomy and in addition for mucinous BOT the appendectomy (Schmalfeldt and Pfisterer, 2007). According to current studies evaluating conservative surgery in this group, high conception rates were achieved after a simple ovarian cystectomy (Longacre *et al*, 2005; Marcickiewicz and Brannstrom, 2006; Tinelli *et al*, 2006; Yinon *et al*, 2007), but the high risk of local recurrence of up to 75% still limited the routine implementation (Suh-Burgmann, 2006). Results of cystectomy for BOT suggest a higher risk of intraoperative cyst rupture and of recurrence when compared with USO or BSO (Poncelet *et al*, 2006; Yokoyama *et al*, 2006). For this reason, ovarian cystectomy or a partial adnexectomy can be performed after a very careful informing of the patient about the recurrence risk and providing that the patient is willing to undergo a careful and prolonged follow-up. Furthermore, the potency of modern possibilities of maintaining fertility should be always discussed (Maltaris *et al*, 2006), whereby cryoconservation of oocytes of patients with malignant and semi-malignant ovarian tumours still remains controversial.

For serous tumours, approximately 40% (range 28–66%), and for mucinous tumours, about 8% (range 0–13%) of the BOTs are observed to be bilateral (Seidman *et al*, 2004; Cadron *et al*, 2007; Kumpulainen *et al*, 2007). In this context, we observed that 19% of the clinical participants performed only unilateral ovarian cystectomy or at least USO. In a case of unilateral adnectomy, a biopsy with histologically negative examination of the contralateral ovary cannot guarantee the detection of possible tumour infiltration and can induce additional damage of the ovarian tissue, so that such an operative procedure seems to be dispensable in many cases. In our survey, the participating clinics indicated a biopsy of the contralateral side in 4–53% of the cases.

The systematic pelvic and para-aortal lymph node dissection, which is generally recommended for patients with ovarian cancer and without post-operative tumour residuals, is however not recommended in BOT. Despite this fact, a removal of enlarged lymph nodes, so called ‘bulky nodes’, can be performed. In contrast to an ovarian carcinoma, invasion in the lymph nodes and metastatic spread of BOT are quite seldom and usually non-invasive (Hart, 2005; Cadron *et al*, 2007; Kumpulainen *et al*, 2007). Moreover, it is not yet clear whether these lymph node implantations represent real metastases, *in situ* transformed secondary muellerian epithelia or hyperplastic mesothelial cells (Shiraki *et al*, 1992; Chang *et al*, 2008). Concerning the performing of lymph node resection, at least 20% of the patients with FIGO stage I have to be upgraded as belonging to FIGO stage IIIc, but the prognostic relevance is in our opinion unimportant (Fauvet *et al*, 2004; Sehouli *et al*, 2005a, 2005b; Silva *et al*, 2006). According to our results, in 9–20% of the evaluated hospitals, a pelvic, and in 3–7%, a para-aortal lymph node dissection were performed. These data are in accordance with other international trials (Fauvet *et al*, 2004; Cusido *et al*, 2007; Kumpulainen *et al*, 2007).

A general recommendation for completion of the hysterectomy cannot be suggested because of lack of a validated benefit for the patients (Fauvet *et al*, 2004; Cadron *et al*, 2007; Cusido *et al*, 2007). On the one hand, this is due to the fact that following conservative surgical management for BOT, the patient outcome is still excellent, but otherwise the rare recurrences usually exhibit a peritoneal location (Seidman *et al*, 1998; Trope *et al*, 2000; Yokoyama *et al*, 2006).

Our survey shows clearly the observation that ‘biological’ status of the patients inside the groups, pre-menopausal, post-menopausal or women in the reproductive age, who desire to preserve fertility, all have a great influence on conservative or radical surgical management. Nonetheless, in the group of patients who wish to preserve their childbearing potential, peritoneal biopsies, omentectomy and also cytological examinations were performed much less often, although all guideline recommendations are clear and these surgical procedures were not found to negatively influence the fertility (Schmalfeldt and Pfisterer, 2007).

### Systemic therapy

So far, there has been no phase III trials performed that explored the role of systemic therapy for patients with BOT. Generally, platin-based chemotherapy regimes were administered among BOT in phase II trials only (Sutton *et al*, 1991; Gershenson *et al*, 1998a, 1998b; Seidman *et al*, 1998; Silva *et al*, 2006). For women with stage I disease only, fewer non-randomised trials were performed, whereby the recurrence rate and patients’ outcome in this group were even more favourable than those of patients without chemotherapy (presumably due to the selection of the patients) (Gershenson *et al*, 1998a, 1998b; Seidman *et al*, 1998; Sehouli *et al*, 2005a, 2005b). This is why the use of adjuvant chemotherapy in patients without post-operative residuals is nowadays not indicated, because treatment results without adjuvant therapy have still been found to be beneficial (Cadron *et al*, 2007; Schmalfeldt and Pfisterer, 2007).

The situation of advanced stage BOT, in which post-operative tumour residuals or invasive implants are present, has to be discussed in a different manner. Borderline tumours of the ovary generally have an excellent prognosis. However, patients with invasive implants and tumour relapse present significantly decreased survival rates. For these ‘high-risk’ patients, various authors recommend platin-based chemotherapy regimes (Gershenson *et al*, 1998a; Seidman *et al*, 1998; Sehouli *et al*, 2005a, 2005b). Some authors recommend – upon detection of an invasive implant – six cycles of platin-based chemotherapy (Gershenson *et al*, 1998b), with 15% response rate within patients with post-operative tumour residual. In our survey, 30% of the participants found no indication for adjuvant treatment for patients with advanced stage BOT. Systemic chemotherapy was preferred by only 0.6% of the clinicians interviewed. Most of them (64%) favoured, however, chemotherapy in complicated disease situations, such as tumour residual, microinvasion or invasive implants. Intraperitoneal chemotherapy is absolutely experimental and should not be applied outside clinical trials. The real effect of systemic chemotherapy on patient outcome for ‘high-risk’ BOT can only be truly assessed in randomised international multicentre studies.

## Conclusion

The results of our multicentre survey underline the high grade of unsureness in the clinical management of BOT in the clinical day. To avoid under- and overtreatment of patients with BOT, educational and training programmes are essential and have to be intensified. Especially, the group of BOT tumours is seen to be optimal for the establishment of a multicentre register. This can also be helpful for a better implementation of evidence-based guidelines.
